# Patterns of uveitis in children according to age: comparison of visual outcomes and complications in a tertiary center

**DOI:** 10.1186/s12886-019-1139-5

**Published:** 2019-06-27

**Authors:** Christiane Al-Haddad, Alaa BouGhannam, Maamoun Abdul Fattah, Hani Tamim, Zeinab El Moussawi, Rola N. Hamam

**Affiliations:** 1 Department of Ophthalmology, American University of Beirut, Beirut, Lebanon; 2 Department of Internal Medicine, American University of Beirut, Beirut, Lebanon; 3 Ocular Immunology and Uveitis- Retina, American University of Beirut Medical Center, PO Box 110236, Beirut, Lebanon

**Keywords:** Amblyopia, Complications, Pediatric, Uveitis

## Abstract

**Background:**

Uveitis in the pediatric population is uncommon, accounting for 2 to 14% of all uveitis cases, yet resulting in significant ocular morbidity. A number of studies have focused on patterns and complications of uveitis in the pediatric age group (≤ 16 years). In this report, we studied children with uveitis syndromes focusing on demographics, anatomic distribution, etiologies, treatment, and complications. We additionally divided subjects into two age groups to look into any differential characteristics pertaining to the younger age group and the role of amblyopia as a cause of visual loss.

**Methods:**

Retrospective chart review of 80 eyes of 49 uveitis patients aged ≤16 years. Subjects were categorized by age of onset into visually immature (≤8 years) and visually mature group (> 8 years). Data compared between the two age groups included demographics, disease characteristics, visual outcomes and complications.

**Results:**

Idiopathic uveitis was the most common diagnosis (51%). Anterior uveitis complications (posterior synechiae and band keratopathy) were more common in the younger group (*p* = 0.002 and *p* = 0.03 respectively) while posterior uveitis manifestations (vitreous haze and vasculitis) were more common in the older age group (*p* = 0.04 and *p* < 0.001 respectively). Amblyopia was the most common cause of vision loss in the visually immature versus cataract in the visually mature.

**Conclusion:**

Anterior uveitis and its complications were more common in visually immature group in our cohort. Amblyopia was identified as the main cause of visual loss in the younger population.

## Background

Uveitis in the pediatric population is uncommon, accounting for 2 to 14% of all uveitis cases, yet resulting in significant ocular morbidity [1, 2].The disease course has a higher rate of complications that can render a young individual legally blind with a lifelong of disabilities [3].Challenges in the management of uveitis in the young population are mainly related to the delay in diagnosis either because of inability to verbalize the complaint, or because the disease itself can be asymptomatic [3, 4]. This is in addition to assessment challenges with poor cooperation in eye examination [5, 6].

Pediatric uveitis has its own specificities related to etiologies like juvenile idiopathic arthritis (JIA) and Kawasaki disease, or subsequent complications like amblyopia. Juvenile idiopathic arthritis is primarily associated with pediatric uveitis and is a main cause for visual loss in children in most of the previous studies [4].Amblyopia due to prolonged inflammation or its sequelae is specific to the pediatric group necessitating early and aggressive intervention.

A number of studies have focused on patterns and complications of uveitis in the pediatric age group (≤ 16 years) [1, 4].In this report, we studied children with uveitis syndromes focusing on demographics, anatomic distribution, etiologies, treatment, and complications. We additionally divided subjects into two age groups to look into any differential characteristics pertaining to the younger age group and the role of amblyopia as a cause of visual loss.

## Methods

All charts of patients presenting to the uveitis clinic at the American University of Beirut Medical Center (AUBMC) - the only tertiary care center for uveitis in Lebanon between January 2009 and January 2013 were reviewed. The study was approved by the American University of Beirut Institutional Review Board and consent was waived. Patients aged ≤16 years at the beginning of uveitis symptoms were identified.

Demographic data pertaining to patients and their disease were collected. These parameters included: age, gender, age at first episode, delay time in referral to our uveitis center (calculated as the time between initial symptoms and first presentation to our center), number of attacks, total time of follow up, number of visits, laterality, chronicity and distribution of the disease, etiology, systemic association, best corrected visual acuity at first and last visit as well as at 6 months and 1 year, intraocular pressure measured by Tonopen or applanation tonometry, different types of ocular and systemic treatments used, ocular surgeries performed and the different types of ocular complications and the date of their occurrence. Complications included cataract, ocular hypertension defined as IOP > 21 mmHg (labeled glaucoma when associated with optic nerve damage), amblyopia – defined as vision worse than 20/30 or 2 lines interocular differences only after affirming control of inflammation and any organic cause of visual loss, cystoid macular edema (CME), posterior synechiae and band keratopathy. Although vasculitis, papillitis and vitreal haze are manifestations of inflammation, we listed them under complications due to their vision threatening risk. We recorded the most significant cause of visual loss for each eye, selecting the more severe cause whenever more than one existed. Uveitis was assessed with a detailed questionnaire on medical history, family history and exposure status and then by a full eye examination and targeted laboratory investigation to diagnose inflammatory and infectious etiologies. All patients are investigated with complete blood count (CBC), Serum Glutamic Pyruvic Transaminase (SGPT), Serum Glutamic Oxaloacetic Transaminase (SGOT), creatinine, urine analysis, Purified Protein Derivative (PPD), Venereal Disease Research Laboratory (VDRL), Treponema Pallidum Hemagglutination Assay (TPHA), and Anti-Nuclear Antibody (ANA). Further investigation is targeted as per history and exam as follows: Infectious etiologies were detected through various tests including PPD, viral antibodies, toxoplasma antibodies, and Polymerase Chain Reaction (PCR). Autoimmune causes were detected by tests investigating Human Leukocyte Antigen (HLA) typing and auto-antibodies such as ANA, anti-double stranded DNA (anti-ds-DNA), cytoplasmic antineutrophil cytoplasmic antibodies (c-ANCA), perinuclear anti-neutrophil cytoplasmic antibodies (p-ANCA), and angiotensin-converting enzyme (ACE). Also brain magnetic resonance imaging (MRI) and radiologic chest imaging were ordered when necessary. Classification of the anatomical location and chronicity of uveitis was based on the International Uveitis Study Group classification system [7]. We also classified patients according to etiology: infectious, autoimmune, or idiopathic. This was confirmed by clinical assessment and/or laboratory and diagnostic tests stated above. Juvenile Idiopathic Arthritis was diagnosed in children according to the International League of Associations for Rheumatology Classification [8]. Behcet disease was diagnosed if the patient fulfilled the criteria of the international study group of Behcet disease [9]. Sarcoidosis diagnosis was confirmed with positive biopsy specimen histological examination, while it was considered as presumed sarcoidosis in case of elevated serum ACE levels and typical granulomatous ocular findings and hilar adenopathy on thin-cut chest computed tomography (CT) scan [10]. Vogt–Koyanagi–Harada (VKH) diagnosis was based on the revised diagnosis criteria for VKH disease [11]. Inflammatory bowel disease (IBD) was diagnosed according to the diagnostic criteria of IBD [12]. Patients with localized retinochoroiditis and a positive toxoplasma antibodies were diagnosed with ocular toxoplasmosis [13]. Patients showing clinical signs of herpetic uveitis such as decreased corneal sensation, herpetic keratitis, increased interocular pressure were diagnosed with Herpes Simplex virus (HSV) [14]. Ocular tuberculosis was diagnosed when patients had positive PPD test, and when uveitis improvement was of 2 grades or more after 2–4 weeks of treatment with anti-tuberculosis drugs [15]. If no identifiable cause of inflammation was found, the term idiopathic uveitis was used.

Given the fact that amblyopia as a cause of visual loss developed only in visually immature children, we further categorized the patients into developing uveitis at age less than or equal to 8 years (visually immature) versus after age 8 years (visually mature) [16]. All the subsequent tabulations followed this categorization and comparison between the two groups was made in all aspects of our analyses. Identification of the main causes of moderate and severe decrease in visual acuity in both visually mature and immature patients was performed. A best corrected vision worse than or equal to 20/50 using Snellen chart was considered a moderate decrease in vision or visual impairment while a best corrected vision worse than or equal to 20/200 was considered to be severe visual loss or legal blindness.

For statistical analysis, we used SPSS v.21 to perform univariate, bivariate, and multivariate analyses. Univariate analysis was performed to compute means and standard deviations of continuous variables and to report a frequency distribution for categorical variables. This was done to assess the characteristics of the population. Bivariate analyses were performed in forms of a chi square test and independent sample t-test to compare the different age groups (≤8 years and > 8 years) with other variables. Statistical significance was set at a *p*-value ≤0.05. As for the multivariate analyses, a binary logistic stepwise regression was performed to identify the predictors of best corrected visual acuity ≤20/50 and ≤ 20/200 at 6 and 12 months. Variables included in this model were age, recorded duration of disease, amblyopia, patching, JIA, corticosteroids treatment, immunomodulatory treatment, biological treatment, retinal complications, cataract, intraocular hypertension, glaucoma, posterior synechiae, and procedures/ surgeries. Also, incidence of vision gain and loss of 2 or more lines per eye-year was calculated.

## Results

Eighty eyes of forty nine patients were enrolled in this study, 32 were visually mature (> 8 years). There was a slight female predominance (55%). The mean age at presentation was 11.4+/− 3.6 years with a mean delay in presentation to our uveitis center of 23.7 months and the mean age at the beginning of the symptoms was 9.1 years. The younger group had a statistically significant longer delay in referral compared to the older group, *p* = 0.004 but also a higher number of follow up. The mean number of uveitis episodes was only 12.6 with no significant difference between the two age groups. Bilaterality was more commonly encountered in the older age group, *p* = 0.63 (Table 1).

**Table 1:** Patient demographic characteristics

	All patients	≤8 years	> 8 years	*p*-value
Number of patients	49	17	32	
Number of eyes	80	27	53	
Number of males (%)	22 (45)	6 (35.3)	16 (50.0)	0.37
Mean age at presentation, years (SD^a^)	11.4 (3.6)	8.5 (3.7)	12.9 (2.6)	< 0.0001
Mean age at beginning of symptoms, years (SD)	9.15 (4.2)	4.3 (2.4)	11.9 (1.9)	< 0.0001
Mean time of uveitis before presentation, months (SD)	23.74 (32.3)	45.1 (40.8)	11.6 (17.7)	0.004
Mean follow up, months (SD)	13.2 (15.1)	15.8 (17.9)	11.9 (13.6)	0.44
Mean number of uveitis episodes (SD)	12.6 (0.4)	15.4 (18.3)	11.2 (13)	0.37
Bilateral, n (%)	31 (63.3)	10 (58.8)	21 (65.6)	0.63
Location, n (%)				
Anterior	20 (40.8)	10 (58.8)	10 (30.3)	0.06
Panuveitis	13 (26.5)	3 (17.6)	10 (31.2)	0.31
Posterior	10 (20.4)	2 (11.8)	8 (25.0)	0.27
Intermediate	6 (12.3)	2 (11.8)	4 (12.1)	1.00
Chronicity, n (%)				
Acute	12 (24.5)	2 (11.8)	10 (30.3)	0.13
Chronic	30 (61.2)	11 (64.7)	19 (59.4)	0.71
Recurrent	7 (14.3)	4 (23.5)	3 (9.1)	0.18

^a^*SD* standard deviation

Anterior uveitis was the most common site of inflammation followed by panuveitis then posterior and finally intermediate uveitis. This pattern was reproduced among the younger patients, who had a greater proportion of anterior uveitis compared to the older group, while the older population had a greater proportion of panuveitis but these findings did not reach statistical significance. Chronic uveitis was the most common across all age groups followed by acute and finally recurrent uveitis (Table 1).

With regards to the different etiologies, idiopathic uveitis was the most common (51%) and no significant differences in etiologies were noted between the younger and older groups (Table 2).

**Table 2:** Etiology of pediatric uveitis stratified by age, *n* (%)

Etiology	All patients *n* = 49	≤8 years *n* = 17	> 8 years *n* = 32	*p*-value
Idiopathic	25 (51.0)	10 (58.8)	15 (46.9)	0.42
Autoimmune	14 (28.6)	4 (23.5)	10 (31.2)	0.57
JIA^a^	6 (12.2)	3 (17.6)	3 (9.4)	0.17
Behcet	3 (6.1)	0 (0)	3 (9.4)	
Sarcoidosis	1 (2.0)	0 (0)	1 (3.1)	
VKH^b^	1 (2.0)	0 (0)	1 (3.1)	
IBD^c^	0 (0)	1 (5.9)	1 (3.1)	
Takayasu	1 (2.0)	0 (0)	1 (3.1)	
TINU^d^	1 (2.0)	0 (0)	1 (3.1)	
Infectious	10 (20.4)	3 (17.6)	7 (21.9)	0.73
Toxoplasmosis	5 (10.2)	2 (11.8)	3 (9.4)	
HSV^e^	2 (4.1)	1 (5.9)	1 (3.1)	
PORN^f^	1 (2.0)	0 (0)	1 (3.1)	
CMV^g^	1 (2.0)	0 (0)	1 (3.1)	
TB^h^	1 (2.0)	0 (0)	1 (3.1)	

*P*-value was not calculated in categories where the etiology was only present in one group

^a^*JIA* Juvenile Idiopathic Arthritis

^b^*VKH* Vogt-Koyanagi-Harada

^c^*IBD* Inflammatory Bowel Disease

^d^*TINU* Tubulu-intestitial nephritis-uveitis

^e^*HSV* Herpes Simplex virus

^f^*PORN* Progressive outer retinalnecrosis

^g^*CMV* Cytomegalovirus

^h^*TB* Tuberculosis

Complications were noted in 78% of the patients presenting to the uveitis clinic, of which 87% were considered sight threatening. If we analyze complications at presentation and until the last visit, we find that the most common was vitreous haze (37.5%). Posterior synechiae and band keratopathy were significantly more prevalent in the younger group. On the other hand, vitreous haze and vasculitis were more significantly encountered in the older population. Amblyopia, as expected, was solely present in the visually immature group with a prevalence of 18.5% (Table 3). Furthermore, 11.25% of the eyes had macular scar, and it was more commonly seen in the visually mature group (67%).

**Table 3:** Eyes with the most common ocular complications stratified by age, n (%)

Complications	All eyes *n* = 80	≤8 years *n* = 27	> 8 years *n* = 53	p-value
Cataract	20 (25.0)	8 (29.6)	12 (22.6)	0.58
Ocular hypertension	15 (18.75)	7 (26.0)	8 (15.0)	0.15
Glaucoma	3 (3.75)	0 (0)	3 (5.7)	< 0.001
Amblyopia	5 (6.3)	5 (18.5)	0 (0)	< 0.003
CME^a^	8 (10.0)	4 (14.8)	4 (7.5)	0.43
Posterior synechiae	26 (32.5)	15 (55.6)	11 (20.7)	0.002
Band keratopathy	10 (12.5)	7 (25.9)	3 (5.7)	0.03
Vasculitis	18 (22.5)	0 (0)	18 (34.0)	< 0.001
Papillitis	20 (25.0)	4 (14.8)	16 (30.2)	0.17
Vitreal haze	30 (37.5)	6 (22.2)	24 (45.3)	0.04

^a^*CME* Cystoid macular edema

No differences in treatment modalities were noticed between groups. Similarly, no significant differences in procedure rates and types were found between the different age categories.

Visual impairment was present in 52% of the visually immature eyes and 37.3% of visually mature eyes at first visit. Changes in visual performance are summarized in Table 5 with the percentage of eyes with visual impairment and legal blindness at 6 months and 1 year. In general there was an improvement in vision at subsequent visits in both age groups. Improvement by 2 or more lines was significant in the younger age group both at 6 and 12 months (*p* = 0.02, 0.03) (Table 4). The incidence of improvement by ≥2 lines was higher in the immature group (0.25 vs 0.18 per eye-year), while the incidence of worsening by ≥2 lines was higher in the mature group (0.13 vs 0.03 per eye-year).

**Table 4:** Visual acuity changes stratified by age, *n* (%)

	All eyes	≤8 years	> 8 years	p-value
At initial visit	*n* = 76	*n* = 25	*n* = 51	
VA^a^ ≤ 20/50	32 (42.1)	13 (52.0)	19 (37.3)	0.22
VA ≤ 20/200	14 (18.4)	7 (28.0)	7 (13.7)	0.13
At 6 months	*n* = 54	*n* = 18	*n* = 36	
VA ≤ 20/50	12 (22.2)	6 (33.3)	6 (16.6)	0.18
VA ≤ 20/200	5 (9.3)	2 (11.1)	3 (8.3)	1.00
Improvement≥2 lines	16 (29.6)	9 (50.0)	7 (19.4)	0.02
Worsening≥2 lines	4 (7.4)	1 (5.6)	3 (8.3)	1.00
At 1 year	n = 49	n = 18	*n* = 31	
VA ≤ 20/50	18 (36.7)	7 (38.9)	11 (35.5)	0.81
VA ≤ 20/200	7 (14.3)	2 (11.1)	5 (16.1)	0.63
Improvement≥2 lines	18 (36.7)	10 (55.6)	8 (25.8)	0.037
Worsening≥2 lines	11 (22.4)	2 (11.1)	9 (29)	0.19
At last visit				
VA ≤ 20/50	24 (31.6)	9 (36.0)	15 (29.4)	0.56
VA ≤ 20/200	10 (13.2)	3 (12.0)	7 (13.7)	0.83
Improvement≥2 lines	17 (22.3)	9 (36.0)	8 (15.7)	0.076
Worsening≥2 lines	7 (9.2)	1 (4.0)	6 (11.8)	0.41

^a^VA: Visual Acuity

**Fig. 1:**
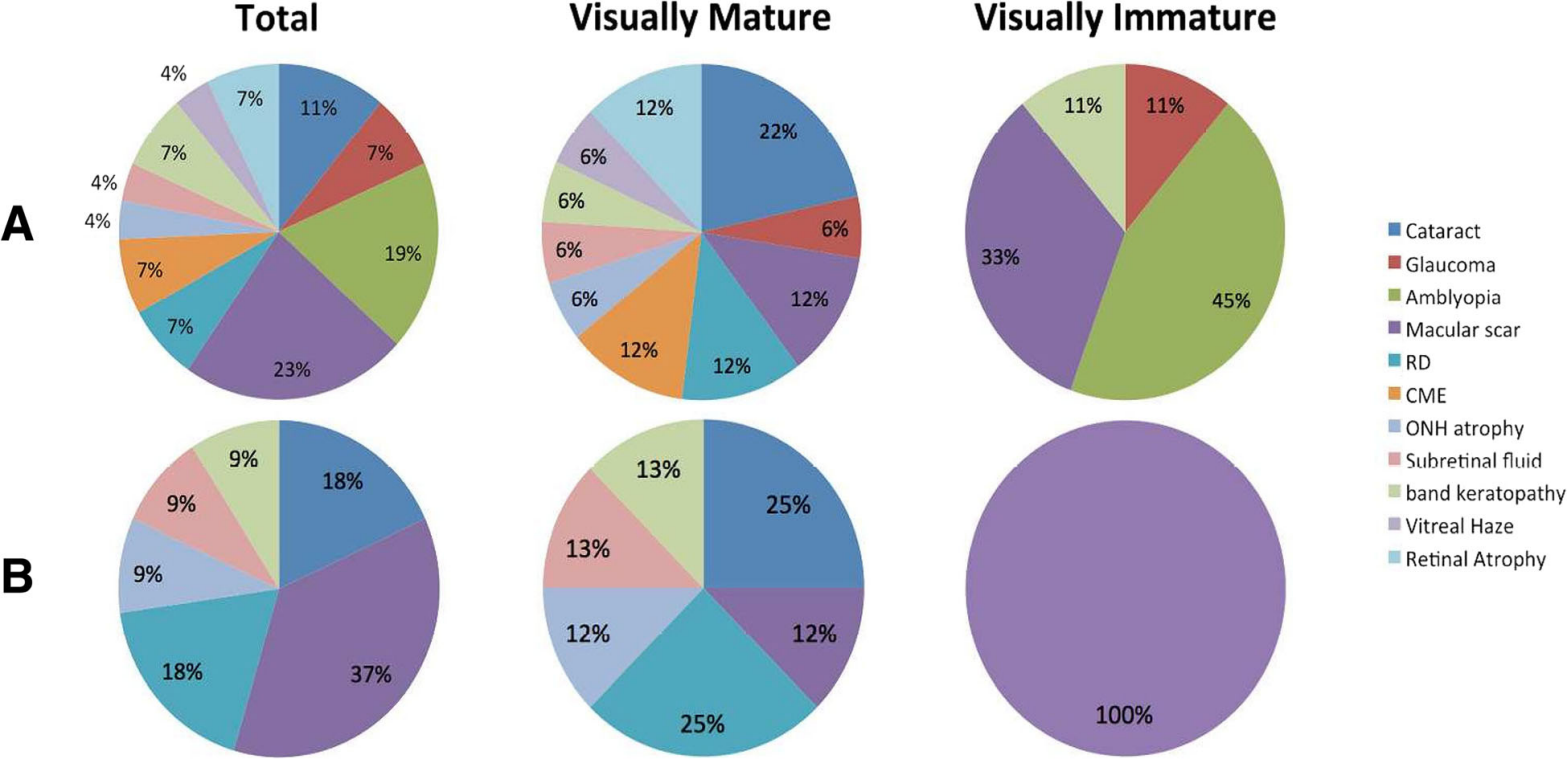
Causes of vision impairment VA ≤ 50 (**a**) and legal blindness VA ≤ 200 (**b**)

The main causes for vision loss were different when compared between the two age groups and classified by the degree of vision disruption. For the visually impaired group (VA ≤ 20/50), the main reason for the drop of vision was macular scar. Amblyopia was the main culprit for visual impairment in the visually immature eyes (44%) while cataract was the main reason in the visually mature group (22%), (Fig. 1a). With respect to severe vision loss (VA ≤ 200), macular scar was the main cause. In visually immature subjects, macular scar was the only cause for VA ≤ 20/200, while in the older group the main causes were cataract and retinal detachment (Fig. 1b).

Multivariate analysis showed that among the different variables studied, only steroid use was a protective factor with an odds ratio of 0.15, but this did not reach statistical significance (*p* = 0.06).

## Discussion

This project was conducted because of the rarity of literature on pediatric uveitis in general and more specifically in Lebanon and the Middle East region. Furthermore, dividing patients into 2 age groups elucidated the significant differences between visually mature and immature eyes, which were not studied previously. In a recent report from our center on the epidemiology of uveitis, we found that the pediatric population accounted for almost 20% of the uveitis clinic patients, which was higher than the percentage reported by other studies [1, 9, 10].

The younger group in our cohort had a higher prevalence of anterior uveitis and suffered from a significant delay in diagnosis when compared to the older group. With an average delay in presentation of 3.5 years in the younger patients, half of these patients had visual impairment at first visit while one third presented with legal blindness which was more than what had been reported in the literature.^5, 7.8,10^

Amblyopia was the most common cause of visual loss in visually immature children while cataract was the main cause in the visually mature group, at a mean duration of uveitis of 23 months.

As for the etiology, we found that idiopathic uveitis prevailed in 51% of the cases in agreement with recent reports (30–55%) [5, 7, 12, 13].All recent studies showed a high prevalence of JIA as the culprit of identifiable causes of uveitis in the pediatric age group (9.4–47%) [5, 12, 13], in agreement with our series (12.2%). Behcet uveitis was the second most prevalent autoimmune cause of uveitis in our sample (6%), which was much higher than the global results (< 2%) [14, 17].This was expected because of the increased prevalence of this disease in countries of the Mediterranean basin [10, 13, 15].Infectious etiologies came third in our study at 20%, generally higher than the worldwide percentage (10%) [5, 16, 18–21],but lower than the prevalence found in studies conducted in the Middle East region (27–33%) [13, 16, 17, 19, 22, 23].Toxoplasmosis was the main cause of infectious uveitis (10%) consistent with recent studies (7.2–25%) [20, 22, 24–27].

There has been a shift in the distribution of the disease since the 1960s; posterior uveitis was more common at that time (22–67% of cases) [14, 20]. Recent reports- including ours- showed that posterior uveitis in the pediatric age group dropped to 5–32%, while anterior uveitis increased to 30–57% [5, 13, 17, 22, 27–29].This change may be possibly referred to better and earlier screening of uveitis in children where this disease was mostly unrecognized or misdiagnosed. Table 5 compares the epidemiology of pediatric uveitis in the Mediterranean basin and the Middle East and North Africa (MENA) region. In general, anterior uveitis and JIA appear to be prevalent in the pediatric population in this region similar to reports from northern Europe and North America [2, 17]. On the other hand, VKH and Behcet’s disease -most commonly described in adults- make it to the top of the list of causes of pediatric uveitis in some reports from Tunisia and Kingdom of Saudi Arabia (KSA).

**Table 5:** Comparison of the epidemiology of uveitis in children in terms of etiology and anatomical classifications in the Mediterranean basin and the MENA region

		Etiology (%)					Anatomical classification (%)			
Country	Sample size	Idiopathic	Auto-immune	Most common autoimmune etiology	Infectious	Most common organism	Anterior	Intermediate	Posterior	Panuveitis
Italy [20] (2009)	257	12.8	55.4	JIA	31	Toxoplasma	47.8	19.4	24.9	7.8
Turkey [30] (2012)	121	16.5	62	Pars planitis	21.5	Toxoplasma	31.4	25.6	24.8	18.2
Tunisia [31] (2005)	64	50	25	Behcet’s and JIA	25	Toxoplasma	31.25	31.25	20.3	17.2
Egypt [32] (2017)	413	28.5	35	JIA	36	presumed parasitic anterior uveitis	27.1	30	18.6	24.2
KSA [33] (2007)	163	5	83	VKH	12	Toxoplasma	42	20	7	31
Lebanon (current study)	50	51	28.6	JIA	20.4	Toxoplasma	40.8	12.3	20.4	26.5

As for the complications, we noticed that anterior segment manifestations/complications (band keratopathy and posterior synechiae) were significantly more common in the younger patients while posterior uveitis complications (vasculitis, vitreous haze, and glaucoma) were more prevalent in the older group (*p* < 0.05). This could be due to anterior uveitis being more common in the younger patients. These findings were consistent with a recent study, which showed an increase in the frequency of posterior and panuveitis and a decrease in that of anterior uveitis with age progression; this could be explained by the higher prevalence of JIA in younger children and toxoplasmosis in adolescents, although these did not reach statistical significance in our study [22]. Furthermore, it is worth noting that 75% of the complications seen in our cohort were present on first visit including 100% of the cataract, amblyopia and band keratopathy. This attests to the need for spreading awareness of the potential complications of this disease and its lifelong toll on the visual performance of these children.

Concerning visual outcomes, the current results showed a more profound visual loss on presentation to the specialist with 18.4% of eyes having legal blindness, 42.1% visual impairment as compared to 9.3–17% and 19–23.6% respectively reported in the literature [11, 18, 34, 35]. These numbers improved to 9.3 and 22.2% respectively after 6 months of management at the specialty clinic, with 29.6% of the eyes gaining at least two lines on the Snellen chart by 1 year reflecting the importance of diagnosing and treating these children promptly. This may reflect the delay in diagnosis and referral of these children to a uveitis specialist in our region with an average delay approaching 2 years in this series (3.5 years in the younger group). We also noted that in the older patients, 11.8% of the eyes lost two or more lines on last visit. This might be due to the progression of cataract in 22% of the eyes.

Amblyopia was the most common cause of visual impairment in the visually immature group. In similar studies looking into complications of pediatric uveitis, amblyopia was sparingly mentioned if at all [1, 5, 6]. Even in the few reports where amblyopia was discussed, it was only in the context of secondary cataract surgery rehabilitation [1]. In our cohort, patients labeled as amblyopic had mostly vision loss due to prolonged inflammation and not because of any complications or surgical interventions. Two patients had structural complications: one had CME that resolved with treatment and another had glaucoma controlled on medications. In the presence of ocular and systemic comorbidities, little attention is usually paid to any amblyopia therapy during the critical time in a child’s development where such treatment would be most beneficial. As such, the protracted course of uveitis per se was the underlying cause of amblyopia in our younger patients who also suffered significant delays in presentation leading to prolonged vision deprivation.

We acknowledge a number of limitations in our work. Conducting this study in a tertiary center for uveitis might have biased the study population to include the more severe cases. Other weaknesses include the small sample size (49 patients, 80 eyes); division of the population into subgroups might have decreased the power of the conclusions. The study is of a retrospective nature, which could increase the chances of lead-time biases. Furthermore, since children with chronic anterior uveitis or intermediate uveitis may remain asymptomatic until the development of complications, some of the children included in the older age group might have onset of disease before age 8. However, it would be impossible to determine the exact start date of uveitis in children with complete certainty, as a significant proportion of uveitis in children is asymptomatic and since young children cannot verbalize their symptoms. As such, we chose the first date of inflammation symptoms reported by parents (based on general ophthalmologist assessment or symptoms) as the first date of uveitis.

## Conclusion

Uveitis in children is a rare disease but can have catastrophic sequelae on the visual outcomes of young patients. This study noted that there was a significant delay in the referral of children especially in the younger age group. This may lead to potentially irreversible complications like undetected amblyopia that was shown to be the most common cause of visual impairment in the visually immature group. Special attention to patch therapy to salvage amblyopic eyes once inflammation subsides should be emphasized in any center providing services to children with uveitis.

## Data Availability

The datasets used and analyzed during the current study are available from the corresponding author on reasonable request.

## Abbreviations

ACE: Angiotensin Converting Enzyme
ANA: Anti-Nuclear Antibody
Anti-ds-DNA: Anti-double stranded DNA
AUBMC: American University of Beirut Medical Center
c-ANCA: Cytoplasmic Anti-Neutrophil Cytoplasmic Antibodies
CBC: Complete Blood Count
CME: Cystoid Macular Edema
CMV: Cytomegalovirus
CT: Computed Tomography
HLA: Human Leukocyte Antigen
HSV: Herpes Simplex Virus
IBD: Inflammatory Bowel Disease
IOP: Itraocular Pressure
JIA: Juvenile Idiopathic Arthritis
KSA: Kingdom of Saudi Arabia
MENA: Middle East and North Africa
MRI: Magnetic Resonance Imaging
p-ANCA: Perinuclear Anti-Neutrophil Cytoplasmic Antibodies
PCR: Polymerase Chain Reaction
PORN: Progressive Outer Retinal Necrosis
PPD: Purified Protein Derivative
SD: Standard Deviation
SGOT: Serum Glutamic Oxaloacetic Transaminase
SGPT: Serum Glutamic Pyruvic Transaminase
TB: Tuberculosis
TINU: Tubulu-Interstitial Nephritis-uveitis
TPHA: Treponema Pallidum Hemagglutination Assay
VA: Visual Acuity
VDRL: Venereal Disease Research Laboratory
VKH: Vogt-Koyanagi-Harada
